# Evaluation of the Influence of Coprophagic Behavior on the Digestibility of Dietary Nutrients and Fecal Fermentation Products in Adult Dogs

**DOI:** 10.3390/vetsci9120686

**Published:** 2022-12-09

**Authors:** Thiago Henrique Annibale Vendramini, Victoria Zavisch Gomes, Gustavo Lima Anastacio, Lucas Ben Fiuza Henríquez, Vanessa Ayumi Ochamotto, Mariana Fragoso Rentas, Rafael Vessecchi Amorim Zafalon, Mariana Pamplona Perini, Pedro Henrique Marchi, Andressa Rodrigues Amaral, Marcio Antonio Brunetto

**Affiliations:** Pet Nutrology Research Center, School of Veterinary Medicine and Animal Science, University of São Paulo, 225, Duque de Caxias Norte Ave, Pirassununga, São Paulo 13635-900, Brazil

**Keywords:** coprophagic, apparent digestibility coefficients, fermentative metabolites

## Abstract

**Simple Summary:**

This study evaluated the influence of coprophagic behavior on the fecal concentrations of lactic acid, short-chain fatty acids (SCFA) and branched fatty acids (BCFA), ammonia nitrogen, fecal pH, and the apparent digestibility coefficients of nutrients and fecal scores of adult dogs.

**Abstract:**

Coprophagia is a common and undesirable behavior observed in dogs; however, little is known about its causes or possible consequences when analysis of the animal’s feces is needed for experimental purposes. Therefore, this study evaluated the effect of coprophagy on digestibility, fecal pH, and fermentative metabolites. Twelve healthy dogs with a mean age of 3.50 ± 1.45 years were included and divided into two groups: coprophagic (COP) and non-coprophagic (NCOP). The study lasted 30 days, the last 6 days being used to collect feces for the analysis of the apparent digestibility of coefficients (ADC), fecal pH, and the concentration of short- and branched-chain fatty acids, ammonia, and fecal lactic acid. Statistical analysis was performed using the SAS software. No differences were observed for most variables, except for the ADC of nitrogen-free extract (NFE), which presented the highest average for the COP. This result should be interpreted with caution, as the NFE is estimated from calculations and was not determined in the laboratory; in addition, the results represent not only starch and sugars but also some parts referring to fibers. Therefore, coprophagy seemed not to influence the fecal variables analyzed.

## 1. Introduction

Coprophagy is a natural phenomenon that consists of the ingestion of feces, performed by several species such as leporids, pikas, primates, marsupials, rodents, and, to a lesser extent, swine, equine, and canine [1,2]. This habit is intrinsically associated with other behavioral and physiological factors and its study is essential for a better understanding of its ecological and biological importance [1].

In rodents, lagomorphs, and perhaps piglets and foals, fecal reingestion provides nutritional benefits as it is a rich source of vitamins, minerals, amino acids, and other nutrients that are not fully digested by the gastrointestinal tract and excreted with the feces, performing a specific digestive function [2]. However, this kind of canine behavior, inherited from their ancestors, is not well understood [3]. One of the few pieces of information available is that during lactation periods, females can present this behavior, due to their instincts concerning hygiene and protection, avoiding possible predators due to the scent of the feces [4].

There are two types of coprophagy: autocoprophagia, the act of ingesting self-produced feces, and alocoprophagia, the act of consuming the feces of other animals, both of which have been observed in dogs [5,6]. Some possible causes for this habit are the presence of one or more cohabiting coprophagic animals, stress, the punishment carried out by a pet’s owners when they see them eating feces, the provision of only one meal a day, and a supply of unbalanced foods, although there is no scientific evidence for the latter [3,5,7].

Coprophagy is a habit commonly observed by dog owners and it is estimated that approximately half of domestic dogs have had at least one episode of coprophagy throughout their lives and approximately 28% demonstrate this habit recurrently, demonstrated in a study by Boze (2008) [8]. According to a pilot study by Chung et al. (2016), among the various canine behavior problems reported by their owners in South Korea, coprophagy was present in 11 of the 174 dogs used in the research, equivalent to 6.3%. This study indicated that possible factors such as obedience training, frequency of walks, and time spent alone at home are associated with behavioral deviations in companion dogs [9].

Thus, many laboratory dogs can present this type of behavior due to stressful situations during their growth and adulthood [10], for example, during periods when they must be isolated and placed in restricted spaces for feces or other types of sample collection. It is also necessary to consider that once an animal in a kennel presents the habit, others can learn from it [7].

Considering this information and that no studies were found in the literature that evaluated the coprophagic behavior under the parameter of nutritional assessment, this study evaluated the possible interference of coprophagy in nutrients’ apparent digestibility coefficients (ADC), fecal pH, and fermentative metabolites in healthy adult dogs.

## 2. Materials and Methods

This study was approved by the Ethics Committee on Animal Use of the School of Veterinary Medicine and Animal Science at the University of São Paulo (CEUA/FMVZ/USP) (1306270821).

### 2.1. Location, Facilities, and Animals

The study was conducted at the Pet Nutrology Research Center (CEPEN-PET) of the School of Veterinary Medicine and Animal Science (FMVZ-USP), located at the Fernando Costa campus in the city of Pirassununga-SP, Brazil.

The animals had their health previously evaluated using physical examination, evaluation of complete blood count, and biomarkers of renal and hepatic function in serum (urea, creatinine, alanine aminotransferase, alkaline phosphatase, cholesterol, triglycerides, and glucose), to exclude possible diseases that could predispose them to coprophagia. Twelve adult dogs that were neutered and aged between 1 and 7 years (3.50 ± 1.45 years) were selected. The animals were separated into two experimental groups: the coprophagic group (COP), composed of six animals from the CEPEN-PET that presented coprophagic behavior, and the non-coprophagic group (NCOP), composed of six owned dogs that did not present coprophagic behavior. All animals had ideal body condition scores (BCSs) (GCOP 5.17 ± 0.41 and GNCOP 4.50 ± 0.55) [11] and ideal muscle mass scores (MMSs) (3/4 points) [12]. The characteristics of dogs regarding their breed, age, and gender per group are presented in Table 1.

The COP animals were housed in collective kennels with dimensions of 3.42 m^2^ of covered area and 7.21 m^2^ of uncovered area (solarium). The NCOP animals stayed at their respective homes. All animals had access to fresh water ad libitum.

### 2.2. Diets and Experimental Design

The design used was completely randomized. During the study, all animals received the same extruded, complete, and balanced dry commercial food (PremieRpet Formula^®^—Adult Dogs/Chicken, Premier pet Indústria e Comércio Ltda, Dourado, Brazil). The chemical compositions and list of ingredients are presented in Table 2. Energy intake for each animal was estimated at 95 kcal per (body weight)^0.75^ a day [13].

The study lasted 30 days: the first 24 days were for diet acclimation and the remaining day were for total fecal sample collection for apparent digestibility and fecal score analyses. On the last day, fresh feces were collected by spontaneous defecation with the help of sterile gloves for fermentation metabolite analysis. All collections of the COP were carried in the Pet Nutrology Research Center. For the NCOP, all owners collected feces in their own houses for apparent digestibility analyses. They received brief training about how to collect feces and store samples. Collections for fermentation metabolites analysis of this group were carried in the Pet Nutrology Research Center, by the researchers responsible for the study.

### 2.3. Apparent Digestibility of Nutrients (ADC) and Fecal Scores

ADC was evaluated using collections of total feces [14]. Food consumption was recorded daily, weighing the amounts offered and refused at each meal. If there was leftover food, the owners of the NCOP animals were instructed to keep them, so that they could be weighed on a semi-precision balance.

Total feces were collected over five days. For NCOP animals, owners were instructed to collect feces after defecation, while for COP animals, these were collected after 24 h of food supply to avoid interfering in the coprophagic behavior. If the animals defecated in the solarium, the feces were transferred to the inside to avoid exposure to sunlight and rain.

The feces were placed in individual plastic bags, identified, and stored in a freezer (−15 °C) for further analysis. At the end of the collection period, stools were thawed, weighed, and homogenized, composing a single sample (fecal pool) per animal. Subsequently, they were dried in a forced ventilation oven (Marconi MA035/2, Piracicaba, Brazil) at 55 °C for 72 h [15]. The pre-dried feces were then grounded in a Willey-type knife mill (Marconi MA340, Piracicaba, Brazil) with a 1mm sieve and then in a micro-knife mill (Marconi MA048, Piracicaba, Brazil). Food samples were grounded in an analytical mill (Ika A11 Basic Mill, Staufen, Germany).

Subsequently, subsamples were taken to determine the contents of dry matter (DM), crude protein (CP), fat by acid hydrolysis (FAH), ash (MM), and crude fiber (CF) from feces and foods and the calcium (Ca) and phosphorus (P) contents according to the methodologies described by the Association of Official Analytical Chemists [15].

CP was estimated from the concentration of organic nitrogen present in the samples. For this analysis, subsamples of 100 micrograms were weighed on an analytical balance, which underwent a digestion process for 2 h, for which 3 mL of sulfuric acid and one gram of a mixture (9:1) of anhydrous sodium sulfate were added in addition to copper sulfate, which served as a catalyst. After this process, 35 mL of distilled water was added and the samples were inserted into the micro-kjedahl still apparatus (Marconi MA036, Piracicaba, Brazil) together with 15mL of 40% sodium hydroxide. Then they were distilled with a 0.02 N hydrochloric acid solution. To calculate the CP concentration, the constant 6.25 was used as the protein molecules contained, on average, 16% nitrogen.

To determine the FAHs of the samples, subsamples of three grams were weighed and subjected to an acid hydrolysis process by boiling them and 75 mL of 40% hydrochloric acid solution for 45 min. Then, the samples were filtered through qualitative filter paper. After drying, they were placed in a Soxhlet extractor device fractionated with six tests (Marconi MA487/6/250, Piracicaba, Brazil) for at least seven hours to be degreased using petroleum ether.

To determine the MM concentrations of the samples, two grams were weighed on an analytical balance, which was later placed in a muffle furnace (Marconi MA385/3, Piracicaba, Brazil) at 500 °C for four hours. After this period, the samples were weighed again and, by difference, the MM value was obtained.

For the quantification of CF, subsamples of 500 micrograms were weighed on an analytical balance, which was placed in specific bags to determine fiber content. After this process, the samples were placed in a fiber digester (Marconi MA444/CI, Piracicaba, Brazil), where they were first subjected to acid digestion with a 1.25% sulfuric acid solution and then to basic digestion with a 1.25% sodium hydroxide solution for 30 min each. After each digestion, three washes were performed with distilled water, each lasting five minutes.

Nitrogen-free extracts (NFE) were calculated by the difference of the sum of the percentages of crude fiber, fat, crude protein, and ash from the total 100%.

Organic matter (OM) was calculated according to the below equation:

OM% = 100 − MM

All analyses were performed in duplicate, except for CF, which was performed in triplicate.

Based on the results obtained in the laboratory, the ADCs of DMs were calculated. After correction for DM, the dietary ADCs of OM, CP, FAH, CF, and NFE were calculated according to the below equation:

ADC% = [nutrient intake (g) − nutrient output (g)]/[nutrient intake (g)] × 100.

Phosphorus contents were determined by the colorimetric method; calcium contents were determined using the titration with EDTA method [15]. Fecal scores were evaluated according to the scale published by the Waltham Research Center [16]. During the period of sample collection for digestibility, values from 0 to 5 were assigned to indicate feces consistency. Values between 2.0 and 2.5 were considered ideal.

### 2.4. Fermentation Metabolites

For the fermentation metabolites analyses, feces were collected in a sterile manner. One sample from each animal was collected to perform all analyses.

For the fecal pH, a benchtop digital pH meter with an autonomous electrode (Starter 3100, pH Bench, Ohaus, São Paulo, Brazil) was used. A total of 1 g of fresh feces with 9 mL of distilled water was homogenized. To measure the pH, the electrode was directly introduced into the 9:1 solution [17].

To analyze the ammonia nitrogen and short- (SCFA) and branched-chain fatty acids (BCFA) content, 3 g of each sample were weighed and mixed with 9mL formic acid at 16% concentration. The mixtures were kept in a refrigerator (5 °C) for seven days and homogenized daily. After this period, the samples were centrifuged in a refrigerated centrifuge (Sorvall Legend MACH 1.6 R, Thermo Fisher Scientific, Waltham, MA, USA) for 15 min at 15 °C at 5000 rpm. This process was repeated three times, the supernatants were extracted, and the sediments were discarded. After extraction, the samples were identified and stored in a freezer (−15 °C).

SCFA and BCFA concentrations were analyzed by gas chromatography [18]. For these analyses, 0.4 mL of the supernatant was transferred to a chromatographic flask, and 0.2 mL of a 3:1 solution of metaphosphoric and formic acid (25% metaphosphoric acid with 98–100% formic acid solution) was added. The internal standard (0.2 mL of 100 mM 2-ethyl-butyric acid) was added to each flask. Subsequently, the supernatant extract (±1.2 mL) of each sample was transferred to chromatographic flasks. Of this extract, 1 μL was injected into a gas chromatograph (GC HP 7890A; Injector HP 7683B, Agilent Technologies, Santa Clara, CA, USA) equipped with an HP-FFAP capillary column (1909F-112; 25 m; 0.32 mm; 0.5 μm; JeW Agilent Technologies, Santa Clara, CA, USA). The injection was performed automatically by the system. The carrier gas used was hydrogen, maintained at a flow rate of 31.35 mL/min. The temperature of the injector and detector was 260 °C. The total chromatographic run time was 16.5 min, divided into three heating cycles: 80 °C, 120 °C, and 205 °C. The fatty acid concentration (mM) was calculated based on an external calibration curve with acetic, propionic, butyric, valeric, isovaleric, and isobutyric acid performed with chromatographic standards [18]. After obtaining the results, they were corrected for mmol/kg DM. To determine the total SCFA, acetic, propionic, and butyric fatty acids results were added, while for the total BCFA, valeric, isovaleric, and isobutyric fatty acids were added. Finally, to determine the total fatty acids, the total SCFA and BCFA values were added.

For fecal ammonia nitrogen concentrations, the extracts were thawed at room temperature and 2 mL of each extract was diluted in 13 mL of distilled water and processed in a micro-Kjeldahl apparatus (Marconi MA036, Piracicaba, Brazil). Distillation was performed with 5 mL of 2N potassium hydroxide solution and titration was performed with 0.005 mol/L hydrochloric acid [19].

Finally, to determine the lactic acid concentration, one gram of feces was weighed, homogenized, and mixed with 2mL of distilled water (1:2 w/v). These mixtures were kept for three days in a refrigerator (5 °C) and homogenized daily. After this period, the samples were centrifuged for 5 min at 2852 rpm (Fanem 206-R Centrifuge Excelsa Baby II, São Paulo, Brazil). The supernatants were extracted and the sediments were discarded. One mL of the supernatant and 6mL of sulfuric acid were placed in a test tube which, after being agitated, were placed in boiling water at 80 °C for three minutes. After the tubes had cooled, 0.1 mL of a solution containing 1.5 g of p-hydroxyphenyl and 100 mL of dimethylformamide was added. The tubes were again placed in boiling water at 80 °C for 90 s. Lactic acid was measured using the spectrophotometric method with absorption at 565 nm (500 to 570 nm) and a reagent blank to calibrate the spectrophotometer (Nova 2000UV Spectrophotometer, Piracicaba, Brazil) [20]. The samples were quantified by comparing them with a 0.08% lactic acid standard.

### 2.5. Statistical Analyses

The normality of the residues was verified by the Shapiro-Wilk test (PROC UNIVARIATE) and the homogeneity of the variances was verified by the Levine test. Analysis of variance was performed by PROC MIXED at 5% significance. Statistical analyses were performed using Statistical Analysis System (SAS Institute Inc., SAS, Cary, NC, USA) software.

## 3. Results

All animals ate the amount of food prescribed and did not lose or gain weight. In addition, all of them remained healthy throughout the study, with no need to exclude any of them. Regarding the fecal production and fermentative metabolites results, no differences were observed between treatments for any variable analyzed (Table 3 and Table 4). With regard to the digestibility results, a difference was observed between the treatments for the ADC of NFE, in which COP presented the highest average.

## 4. Discussion

Coprophagic behavior in laboratory dogs has already been reported by different researchers; in some cases, the habit was frequent, and in others, it was sporadic [21,22]. Similar findings have been observed in owned dogs [3,7,23]. However, few studies have evaluated the effect of this behavior according to different fecal variables in dogs. According to Nijsse et al. [23], coprophagy in dogs can interfere with the analysis of parasitic infection in feces, leading to overestimated results.

In this study, no differences were observed for the fermentation metabolites variables, fecal pHs, total fecal productions, fecal scores, and most ADCs, except for NFE, which had the highest mean for COP. However, this result should be interpreted with caution as although NFE is frequently used in dogs’ nutrition, this variable was estimated by an equation [24]. In addition, it was determined after obtaining the results for MM, CP, FHA, and CF, so it incorporates errors from the previous analysis [24,25].

The crude fiber analysis has an important limitation since it only analyzed the amount of fiber within the sample, reducing the soluble fractions of the sample and, partially, the insoluble ones [26]. This technique deficiency was confirmed in the evaluation of food for dogs and cats by Oliveira et al. [27], in which dry extruded cat and dog foods were evaluated for fiber composition and digestibility, comparing total dietary fiber, neutral detergent fiber, acid detergent fiber, and crude fiber. The results showed that crude fiber analysis did not correlate with any other method. Another study [28] evaluated the differences between the maximum concentrations of crude fiber and total concentrations of dietary fiber (which would be the gold standard method today) in dry and wet supporting foods for dogs. The authors concluded that in the absence of information on total dietary fiber concentration, crude fiber content is not a good reference to evaluate the fiber content of a diet for dogs.

This is justified by the fact that it does not separate cellulose from hemicellulose and solubilize part of the lignin and hemicellulose. This method provides values that can change due to the use of drastic digestion, which leads to the loss of some components [26], and, therefore, the values and digestibilities obtained in our study, which included crude fiber in their estimations, may not be substantially reliable. Thus, the estimated NFEs may not be just starch and sugars but the sum of these with alkali-soluble hemicellulose and lignin and pectin, which are part of the fiber group [25,29].

In a study conducted by Ramos et al. [30] with dogs, the fecal production and ADCs of DM, OM, CP, FAH, and gross energy were compared between two groups: with and without environmental enrichment. It was observed that the non-enrichment group had sporadic cases of coprophagic behavior and that providing enrichment reduced this habit. When comparing the ADCs of the groups, the authors did not observe any differences. However, as opposed to this research, in the present study, cases of coprophagia were frequent in the COP group.

The results found in our study may be of great importance to the scientific community, as there is a question of whether the habit of coprophagy could interfere with some results of scientific studies, especially those related to the effects of food on fecal variables, such as those analyzed in the present study. The fact that the animals consumed part of the feces produced during the collection period has led some authors to question whether this may have caused underestimations of fecal production, resulting in consequent overestimations of the apparent digestibility of the nutrients in addition to interfering in the concentrations of almost all the metabolites found in the feces. This issue is particularly important since coprophagy behavior has been observed with high frequency, including in laboratory dogs [10], which can be attributed to stressful situations, including feces collection periods during an experiment, where animals must be separated and are often trapped in metabolic cages.

In addition, given the current knowledge about the importance of intestinal health for the whole organism, which reflects the general health of an individual, research evaluating the effects of different nutritional components on intestinal health has intensified in the last decade [31,32,33,34,35,36,37,38,39,40,41,42,43,44,45]; therefore, it was of paramount importance that the possible effects of coprophagy on these variables were investigated to assist researchers in choosing the most appropriate collection methods and ensure the accurate interpretation of results related to the variables measured in the present study.

Meyer et al. [46] have cited diseases such as exocrine pancreas insufficiency as predisposing causes of coprophagy [47]. In this study, only healthy coprophagic animals were included. For this, physical examinations and blood collection for blood count and biochemical analysis were performed to ensure that the results found were only related to coprophagia and not to other factors such as diseases.

It was not possible, however, to standardize dogs’ age, sex, and breed. However, other authors have reported that these characteristics do not appear to be related to coprophagy [2,7].

A limitation of this study was the number of animals used in each group. The experimental design used was completely randomized, which caused variation, treatment effects, and residue. If a difference between treatments exists, it is more difficult to find when the number of experimental units per treatment is small [48]. However, this was the first study on the subject. The results found can serve as a basis for comparison with future research results with a greater number of experimental units per treatment which evaluate the same variables studied here.

## 5. Conclusions

According to the results obtained in this experiment, coprophagic behavior did not result in differences in the concentrations of fecal fermentation products evaluated (lactic acid, ammonia, and short- and branched fatty acids), fecal pH, and apparent nutrient digestibility coefficients (except nitrogen-free extract) compared to the control group. These results are valuable in the field of small animal nutrition as coprophagy is frequent in laboratory dogs undergoing research related to the effects of nutritional additives on intestinal health. Therefore, it was of paramount importance that the possible influence of coprophagy in fecal variables was elucidated. It is noteworthy that the results of the present study indicate that the occurrence of coprophagy in laboratory dogs does not need to be taken into account when carrying out the experiment design. Additionally, the interpretation of the results was related to the fecal variables analyzed in the present study and future studies are needed to investigate the effects of coprophagy on other related variables, such as the fecal microbiota.

## Figures and Tables

**Table 1:** Breed, age, and gender per group.

NCOP		COP	
Breed	Age	Breed	Age
Yorkshire terrier (M)	5 years	Beagle (M)	3 years
Border collie (M)	4 years	Beagle (M)	3 years
Mixed breed (F)	4 years	Beagle (F)	3 years
English cocker spaniel (F)	2 years	English cocker spaniel (M)	3 years
Mixed breed (M)	4 years	English cocker spaniel (F)	2 years
Akita Inu (F)	7 years	Labrador retriever (M)	2 years
Mean ± SD	4.33 ± 1.63	Mean ± SD	2.67 ± 0.52

Legend: NCOP = non-coprophagic; COP = coprophagic; (M) = male; (F) = female; SD = standard deviation.

**Table 2:** Chemical compositions of and ingredients in the food ^1^.

Item	%
Dry matter (%)	91.82
Chemical composition in dry matter	
Organic matter	93.95
Crude protein	30.09
Fat	19.61
Ash	6.05
Crude fiber	7.59
Nitrogen-free extract	36.66
Calcium	1.25
Phosphorus	0.86

^1^ Poultry viscera meal, corn gluten 60, dehydrated egg, pig protein isolate, whole grain corn, brewer’s rice, beet pulp, poultry fat, fish oil, swine fat, propionic acid, BHA and BHT, potassium chloride, sodium chloride, pig and poultry hydrolyzate, dry brewer’s yeast, mannanoligosaccharides (0.20%), yeast cell wall, vitamin A, vitamin B12, vitamin C, vitamin D3, vitamin E, vitamin K3, folic acid, pantothenic acid, biotin, choline chloride, niacin, pyridoxine, riboflavin, thiamine, amino acid chelated copper, amino acid chelated iron, potassium iodide, amino acid chelated manganese, selenium proteinate, copper sulfate, iron sulfate, manganese sulfate, zinc sulfate, amino acid chelate zinc. Metabolizable energy = 4040 kcal/kg.

**Table 3:** Apparent nutrient digestibility coefficients, fecal scores, and total feces productions of the experimental treatments.

Item	Treatments		SEM	*p*
	NCOP	COP		
Apparent digestibility coefficients (%)				
Dry matter	83.97	86.80	1.172	0.1186
Crude protein	87.25	89.14	0.988	0.2053
Fat	97.45	97.35	0.258	0.7834
Crude fiber	73.96	78.41	2.224	0.1880
Ash	23.97	33.50	5.605	0.2568
Nitrogen-free extract	86.49	90.13	1.095	0.0408
Organic matter	87.83	90.23	0.927	0.0975
Fecal production				
Fecal score	2.40	2.43	0.064	0.3229
Fecal production (g/day/DM)	24.65	24.19	1.679	0.6126

Legend: NCOP = non-coprophagic; COP = coprophagic; SEM = standard error of the mean; DM = dry matter.

**Table 4:** Fecal pH, lactic acid, ammonia, and short- and branched-chain fatty acid concentrations of the experimental treatments.

Item	Treatments		SEM	*p*
	NCOP	COP		
Fecal pH	6.90	6.83	0.07	0.5230
Latic acid (mMol/kg DM)	50.05	71.11	9.117	0.1334
Ammonia (mMol/kg DM)	105.09	113.57	11.168	0.6031
Total FA (mMol/kg DM)	106.56	119.40	10.140	0.3917
Short-chain fatty acids, mmol/Kg DM				
Acetic acid	59.37	68.09	5.949	0.3243
Propionic acid	27.49	28.03	5.324	0.9444
Butyric acid	13.25	17.34	1.674	0.1146
Total SCFA	100.12	113.47	9.827	0.3597
Branched-chain fatty acids, mmol/Kg DM				
Valeric acid	0.37	0.27	0.128	0.6047
Isovaleric acid	2.69	2.15	0.347	0.2906
Isobutyric acid	3.36	3.49	0.416	0.8287
Total BCFA	6.44	5.92	0.594	0.5542

Legend: NCOP = non-coprophagic; COP = coprophagic; SEM = standard error of mean; DM = dry matter; FA = fatty acids; SCFA = short-chain fatty acids; BCFA = branched-chain fatty acids.

## Data Availability

Not applicable.
